# CEST MRI detectable liposomal hydrogels for multiparametric monitoring in the brain at 3T

**DOI:** 10.7150/thno.40146

**Published:** 2020-01-12

**Authors:** Xiongqi Han, Jianpan Huang, Anthea K.W. To, Joseph H.C. Lai, Peng Xiao, Ed X. Wu, Jiadi Xu, Kannie W.Y. Chan

**Affiliations:** 1 Department of Biomedical Engineering, City University of Hong Kong, Kowloon Tong, Hong Kong;; 2 City University of Hong Kong Shenzhen Research Institute, Shenzhen, China;; 3 Russell H. Morgan Department of Radiology and Radiological Science, The Johns Hopkins University School of Medicine, Baltimore, USA;; 4 Department of Electrical and Electronic Engineering, The University of Hong Kong, Pokfulam, Hong Kong.; 5 F.M. Kirby Research Center for Functional Brain Imaging, Kennedy Krieger Institute, Baltimore, USA.

**Keywords:** CEST MRI, hydrogel, liposome, glioblastoma

## Abstract

Adjuvant treatment using local drug delivery is applied in treating glioblastoma multiforme (GBM) after tumor resection. However, there are no non-invasive imaging techniques available for tracking the compositional changes of hydrogel-based drug treatment.

**Methods**: We developed Chemical Exchange Saturation Transfer Magnetic Resonance Imaging (CEST MRI) detectable and injectable liposomal hydrogel to monitor these events *in vivo* at 3T clinical field. Mechanical attributes of these hydrogels and their *in vitro* and *in vivo* CEST imaging properties were systematically studied.

**Results**: The MRI detectable hydrogels were capable of generating multiparametric readouts for monitoring specific components of the hydrogel matrix simultaneously and independently. Herein, we report, for the first time, CEST contrast at -3.4 ppm provides an estimated number of liposomes and CEST contrast at 5 ppm provides an estimated amount of encapsulated drug. CEST contrast decreased by 1.57% at 5 ppm, while the contrast at -3.4 ppm remained constant over 3 d *in vivo*, demonstrating different release kinetics of these components from the hydrogel matrix*.* Furthermore, histology analysis confirmed that the CEST contrast at -3.4 ppm was associated with liposome concentrations.

**Conclusion**: This multiparametric CEST imaging of individual compositional changes in liposomal hydrogels, formulated with clinical-grade materials at 3T and described in this study, has the potential to facilitate the refinement of adjuvant treatment for GBM.

## Introduction

Brain cancer is a devastating disease, and glioblastoma multiforme (GBM) is the most aggressive brain tumors with a median survival of 12-15 months 1-3. Currently, the standard treatment for GBM is maximal surgical resection followed by adjuvant radiation and chemotherapy, including temozolomide (TMZ) and carmustine (BCNU) 4. Nevertheless, about 90% of patients have tumor recurrence within two years 5. As an alternative therapy, Gliadel^®^ - a carmustine wafer implanted into the tumor resection cavity is the only local treatment approved by FDA for newly-diagnosed and recurrent GBM 6. However, its clinical application has been hampered by limited drug penetration, incomplete coverage, and edema related to wafer degradation 7-9.

While many hydrogel-based drug delivery systems have been developed for local treatment, a noninvasive imaging technique for monitoring multi-components of the hydrogel matrix after transplantation is lacking. Magnetic resonance imaging (MRI) is a versatile imaging modality featured by excellent soft-tissue contrast and without imaging depth limitation or need for radioactive tracers 10-13. In particular, Chemical Exchange Saturation Transfer (CEST) MRI can sensitively image both endogenous and exogenous molecules non-invasively by detecting natural exchangeable protons on molecules 14-16. It does not require metallic contrast agents, which could lead to nephrogenic systematic fibrosis 17. Many preclinical theranostic applications have demonstrated the uniqueness of CEST MRI in imaging of proteins, drug delivery, and non-invasive hydrogel degradation 18-28. We and others have developed various CEST-detectable liposomes (LipoCEST) 25-27, 29-35, which enhance the sensitivity of CEST *in vivo* by increasing local concentrations of exchangeable protons. It is sensitive enough to probe cell viability by sensing local pH changes in alginate microcapsules, a pioneering application of CEST MRI in monitoring hydrogel-based therapy 25. Besides these contrasts detected at the positive frequency offsets from water, researchers are exploiting other exchangeable protons at the negative offset frequencies; for example, Nuclear Overhauser Enhancement (NOE) for aliphatic protons 15, 36-38 has been applied to study endogenous proteins or lipids *in vivo* 35, 36. These positive and negative offsets of CEST contrast enable the monitoring of multiple processes *in vivo*, which is advantageous for revealing compositional changes in hydrogel-based therapies.

The unique properties of injectable hydrogels could address the drawbacks of carmustine wafer in brain tumor treatment 39-41. The hydrogel is a three-dimensional hydrophilic network with tunable mechanical properties for delivering drugs or cells. In particular injectable hydrogels can be applied *via* minimally invasive procedures 42-44. Alginate (Alg) and hyaluronic acid (HA) are the most widely used natural biocompatible polymers in clinical applications and readily form hydrogels by crosslinking with divalent cations (e.g., Ca^2+^, Ba^2+^) and methylcellulose (MC), respectively 44-50. The hardness of hydrogel is another important factor to consider for applications in the brain to minimize the risk of tumor recurrence. It has been reported that relatively soft hydrogels could deter tumor cell proliferation and migration 51, 52. Both alginate and hyaluronic acid methylcellulose (HAMC) typically form hydrogels with storage modulus at a range of 10-1000 Pa and are regarded as soft type hydrogel 53, 54. Moreover, the hydrogel matrix should be capable of carrying a variety of drugs and amenable to tailoring for controlled drug release 55, 56. To further support sustainable release, drug-loaded liposomes can be incorporated into the hydrogel matrix. The liposomes are the first FDA approved versatile nanocarriers 56, 57, which are composed of a phospholipid bilayer and an aqueous core for hydrophobic and hydrophilic drug loading 58, 59. For example, the burst release of drugs from liposomes could be minimized in hydrogel 39, 40, 60, 61.

We have previously shown that barbituric acid (BA)-loaded liposomes with contrast at 5 ppm away from water could be used to monitor the delivery of a liposomal drug (Doxil^TM^) to tumors and of mucus-penetrating particles to mucus-covered tissues 26, 27. In this study, we have developed a newly designed MRI-detectable liposomal hydrogel based on clinical-grade biopolymers to enable multiparametric imaging to guide local treatment in the brain. It composed of BA liposomes (BAL) in either alginate or HAMC hydrogels. These hydrogel matrices were designed to be injectable, soft, and detectable by CEST MRI, especially with distinctive CEST contrast at the common 3T clinical field. The designated CEST contrast could measure the amounts of intraliposomal drugs and the liposome nanocarrier. Furthermore, the hydrogels had CEST contrast at frequency offsets that were widely separated to facilitate multiparametric imaging *in vivo* at 3T. One of the major hurdles of multiparametric CEST MRI at 3T is the small peak separation (1-2 ppm) of the corresponding CEST contrast. Our design could overcome the drawbacks in imaging multi-components in the hydrogel matrix at 3T and provide a theranostic approach for adjuvant treatment in brain cancer. These unique CEST and rheological properties are versatile for noninvasively and longitudinally monitoring hydrogel-based therapies in GBM treatment.

## Results

### Mechanical studies of hydrogels

We formulated five liposomal hydrogels based on alginate and HAMC. Their rheological and viscoelastic properties were examined with the goal of producing injectable and mechanically soft hydrogels that would not favor tumor cell proliferation or migration 51, 52. Alginate is composed of (1,4)-linked β-D-mannuronate (M) and α-L-guluronate (G) residues. We chose alginate with 60% of G residues for all our formulations. Alginate hydrogel formulations were prepared using different alginate concentrations (i.e., 1% or 2% alginate) and crosslinking densities defined by the ratio of G components to calcium ions (i.e., 40% or 80% crosslinking) with or without the addition of liposomes. All resulting formulations showed soft hydrogel properties with storage modulus at 10-250 Pa.

**Figure 1:**
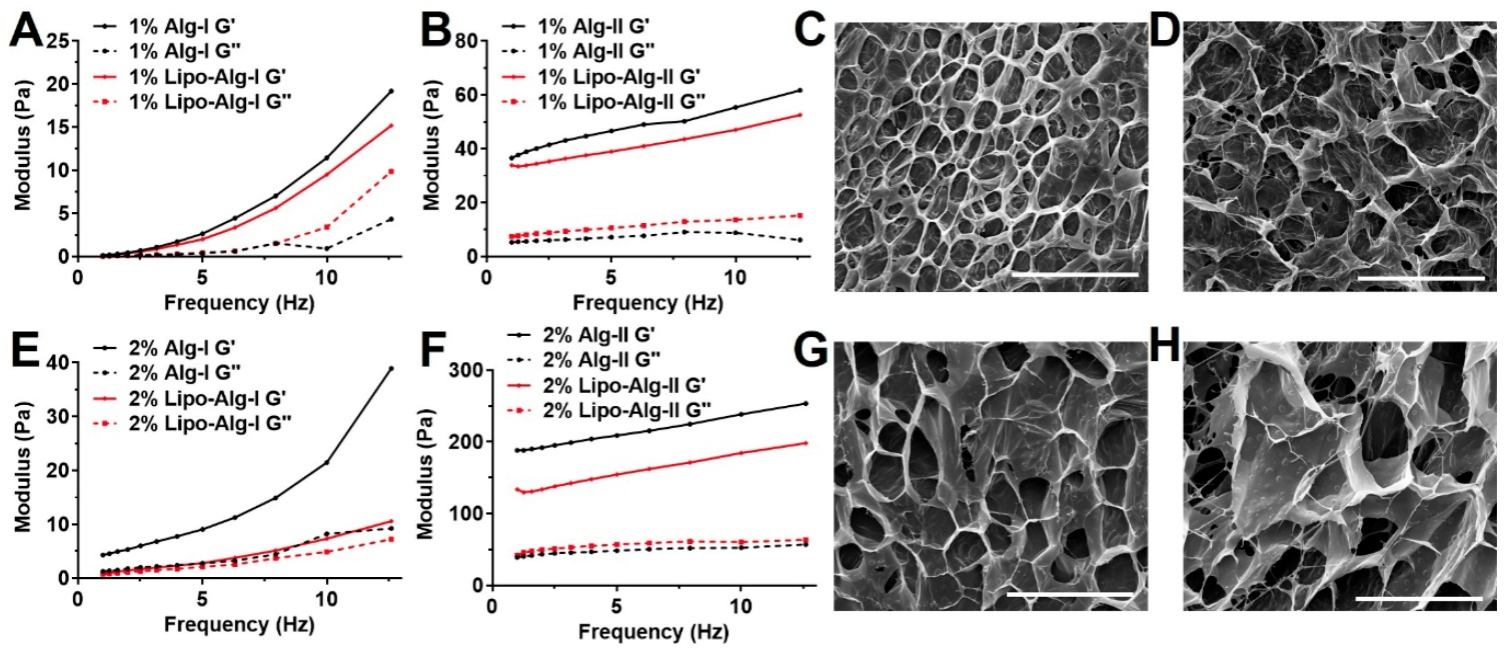
Frequency sweep measurements and scanning electron microscopy (SEM) images of alginate hydrogel. **(A)**, **(B)** and **(C)**, **(D)** are 1 wt% alginate hydrogels with 40% and 80% crosslinking; **(E)**, **(F)** and **(G)**, **(H)** are 2 wt% alginate hydrogels with 40% and 80% crosslinking. Ag-I and Ag-II are alginate hydrogels with 40% and 80% crosslinking, respectively. The measurements for each sample were performed three times. Scale bar = 200 μm.

As shown in Figure 1, the storage modulus (G') in all hydrogels was higher than loss modulus (G'') in full frequency window, indicating the hydrogel status. The frequency dependency of hydrogel demonstrated the formation of viscoelastic networks in the hydrogel 62. Both G' and G'' were highly dependent on crosslinking density and alginate concentration. Hydrogel with 80% crosslinking density was less dependent on frequency compared with 40% crosslinking density, suggesting the relative stable hydrogel networks. As alginate concentration and crosslinking density increased from 40% to 80%, G' (at 10 Hz) increased from 11.4 ± 3.2 (1%) and 21.5 ± 5.2 (2%) to 55.4 ± 7.5 (1%) and 238.5 ± 18.7 (2%). This increase in crosslinking density has resulted in a large increase in G', while the addition of liposomes has a relatively less effect on G'. The mechanical property and porosity were comparable among these formulations of hydrogels with or without liposomes, which are regarded as soft type of hydrogels with G' less than 300 Pa 51,52.

All formulations showed viscosities less than 5 Pa·s (**Figure S1 A** and** B**), which further decreased to a much smaller value (<1 Pa·s) under higher shear rate (>30 s^-1^), indicating their superior injectability. The viscosities of all hydrogels decreased with increments in the shear rate, displaying a shear-thinning behavior as the hydrogel networks were perturbed by the shear. Also, SEM images (**Figure 1** and **Figure S2**) showed that all hydrogels had well-defined and microporous structures. The alginate hydrogel showed pore size in the range of 30-60 μm (**Figure 1**), while the liposomal hydrogel showed a slightly larger pore size of about 90 μm (**Figure S2**).

**Figure 2:**
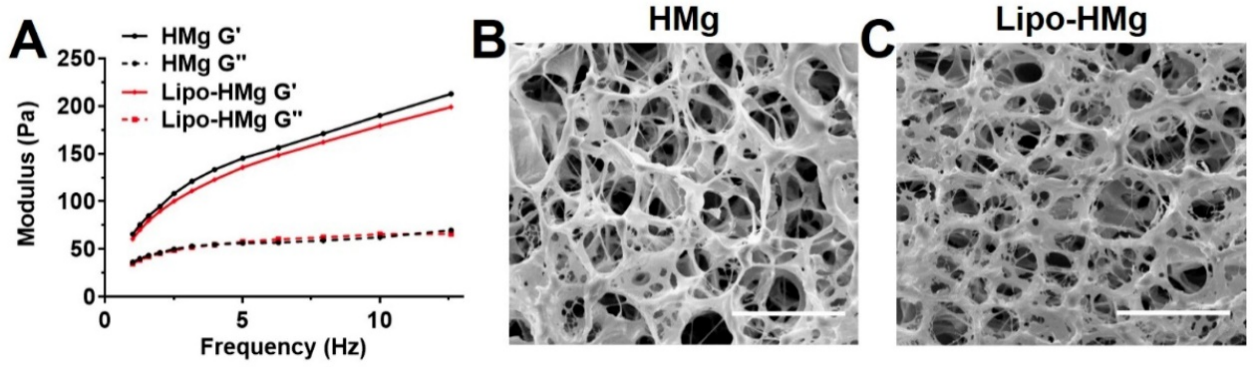
Frequency sweep measurements and scanning electron microscopy (SEM) images of HAMC hydrogel.** (A)** frequency sweep measurements.** (B)** and **(C)** are representative morphologies of HAMC hydrogel without and with liposomes. The measurements for each sample were performed 3 times. Scale bar = 50 μm.

Both HAMC formulations with and without liposomes resulted in a soft type of hydrogels with G' in the range of 170 to 200 Pa (**Figure 2 A**) at 10 Hz. The addition of liposomes resulted in a slight decrease in G' by 5.6%. This finding was consistent with those of the alginate hydrogel formulations, indicating the addition of liposomes slightly decreased the storage modulus of hydrogel matrices. Both hydrogels showed shear-thinning behaviors (**Figure S1 C**), and under high shear rate (>10 s^-1^), the viscosities were less than 5 Pa·s indicating favorable injectability. The SEM images of HAMC hydrogels, as displayed in **Figure 2 B** and **C**, showed a highly porous structure**.** The pore size of HAMC hydrogels with and without liposomes was similar (around 10 μm) and was smaller than that of the alginate hydrogels. The addition of liposomes did not increase the pore size, as observed in the case of alginate.

We found that the viability of brain cancer cells in the liposomal hydrogel formulations was compromised as compared to those without liposomes (**Figure S3B**). In particular, the viability of tumor cells in the liposomal hydrogel formulations of 1% alginate at 80% crosslinking (1% Lipo-Alg-II) was significantly lower (p<0.05, n=8) than that without liposomes (1% Alg-II). Similarly, other liposomal hydrogel formulations (2% Lipo-Alg-I, 2% Lipo-Alg-II, and 0.75% Lipo-HMg) showed a slightly lower tumor cell viability as compared to those formulations without liposomes (n=8) (**Figure S3**). Notably, the storage modulus was slightly lower in the presence of liposomes (**Figure 1** and **2**). Both kinds of hydrogels were soft, with G' (at 10 Hz) in the range of 10-300 Pa. As for the rheological properties to enhance the treatment efficacy 51, 52, the porous hydrogel with low tumor cell viability and injectability in a 10-μl syringe, i.e., 2% alginate and 40% crosslinked hydrogel was chosen for further study.

### Physiochemical and CEST properties of BAL

**Figure 3:**
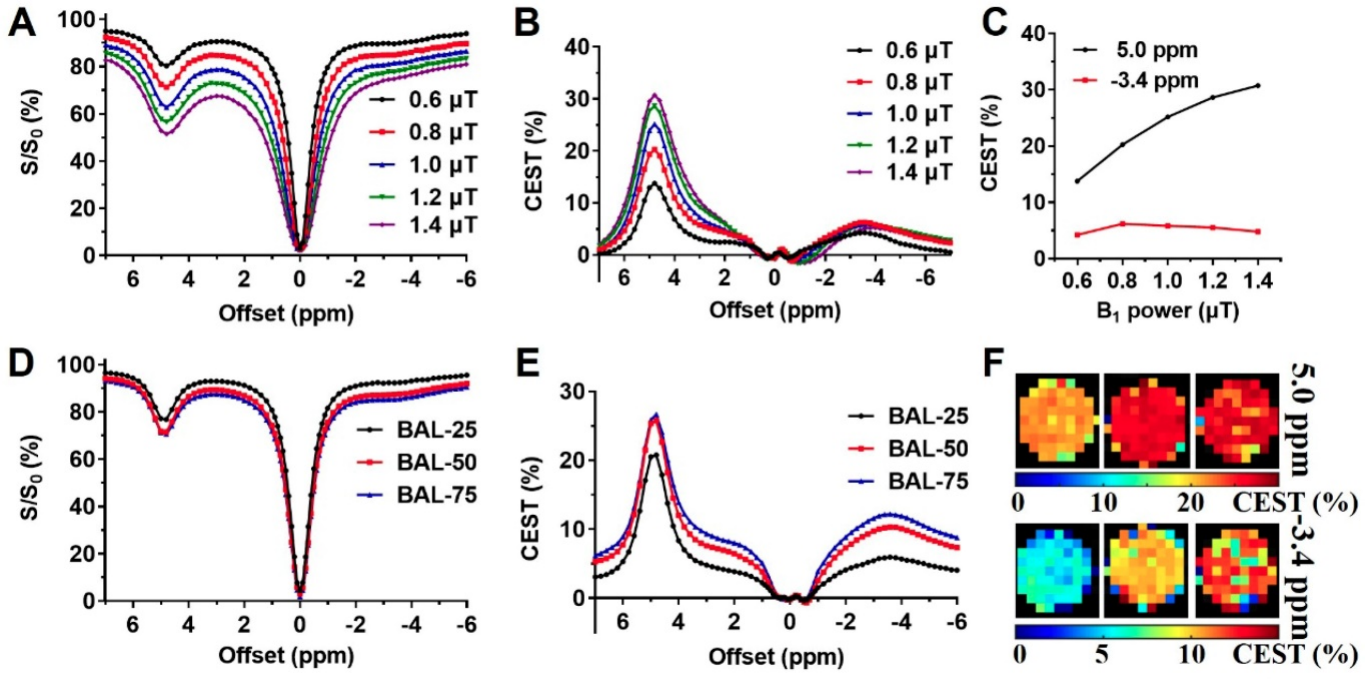
B_1_ optimization and CEST properties of BA-liposomes (BAL, n=3). **(A), (B),** and** (C)** are Z-spectra and corresponding CEST contrasts of BAL (with 75 mg/mL lipids) under various B_1_ powers; **(D)**, **(E)** and** (F)** are Z-spectra, corresponding CEST contrast, and maps at 5 ppm and -3.4 ppm of various BAL formulations.

Liposomes are known to enhance drug delivery; however, imaging the number of liposomes is challenging. We first examined BAL at different concentrations at 3T using CEST MRI and observed uniform distribution with a size of about 200 nm, PDI at around 0.2, and near-neutral surface charge (**Table 1**). The BA concentration in the final liposome solution increased with lipid concentration, from 13.57 to 19.21 mg/mL (**Table 1**). Moreover, the particle concentration also increased with increased concentration. At 25 mg/mL lipid, the particle concentration was 1.0×10^16^, while at 75 mg/mL lipid, the particle concentration increased to 1.7×10^16^ (**Table S1**). Interestingly, in addition to the CEST contrast of BA at 5.0 ppm, BAL also showed a distinctive CEST contrast at -3.4 ppm (**Figure 3**) produced by lipid composition. With these distinctive contrasts, the amount of intraliposomal drug (BA) and the number of liposomes can be simultaneously measured *in vitro*.

To optimize the multiparametric imaging of intraliposomal drug and liposomes, we tested the saturation parameter (B_1_) from 0.6 to 1.4 μT at pH 7.0 *in vitro* using liposomes with lipid concentration of 75 mg/mL. As shown in Figure 3 A, B, and C, the CEST contrast at 5 ppm increased almost linearly with B_1_ field strength. While the CEST contrast at -3.4 ppm showed the highest contrast at 0.8 μT (**Figure 3 C**), further increasing the B_1_ field led to a decrease. Thus, 0.8 μT was selected for the following *in vitro* studies.

With the increase in BAL concentration, CEST contrast (**Figure 3 D, E,** and **F**) increased from 20.8% and 25.8% to 26.7% at 5.0 ppm, and from 5.9% and 10.3% to 13.2% at -3.4 ppm, as listed in **Table S1**. Also, CEST contrast at 5 ppm increased by 5.9% as BA concentration increased from 13.6 to 19.2 mg/ml, and CEST contrast at -3.4 ppm increased by 7.3% as liposome concentrations increased from 1.0×10^16^ to 1.7×10^16^ particle/mL. Both CEST contrasts at 5 ppm and -3.4 ppm showed an approximate linear correlation with their respective concentrations (**Figure S4**, R^2^> 0.96) within the tested range.

### *In vitro* CEST properties of the liposomal hydrogel

**Figure 4:**
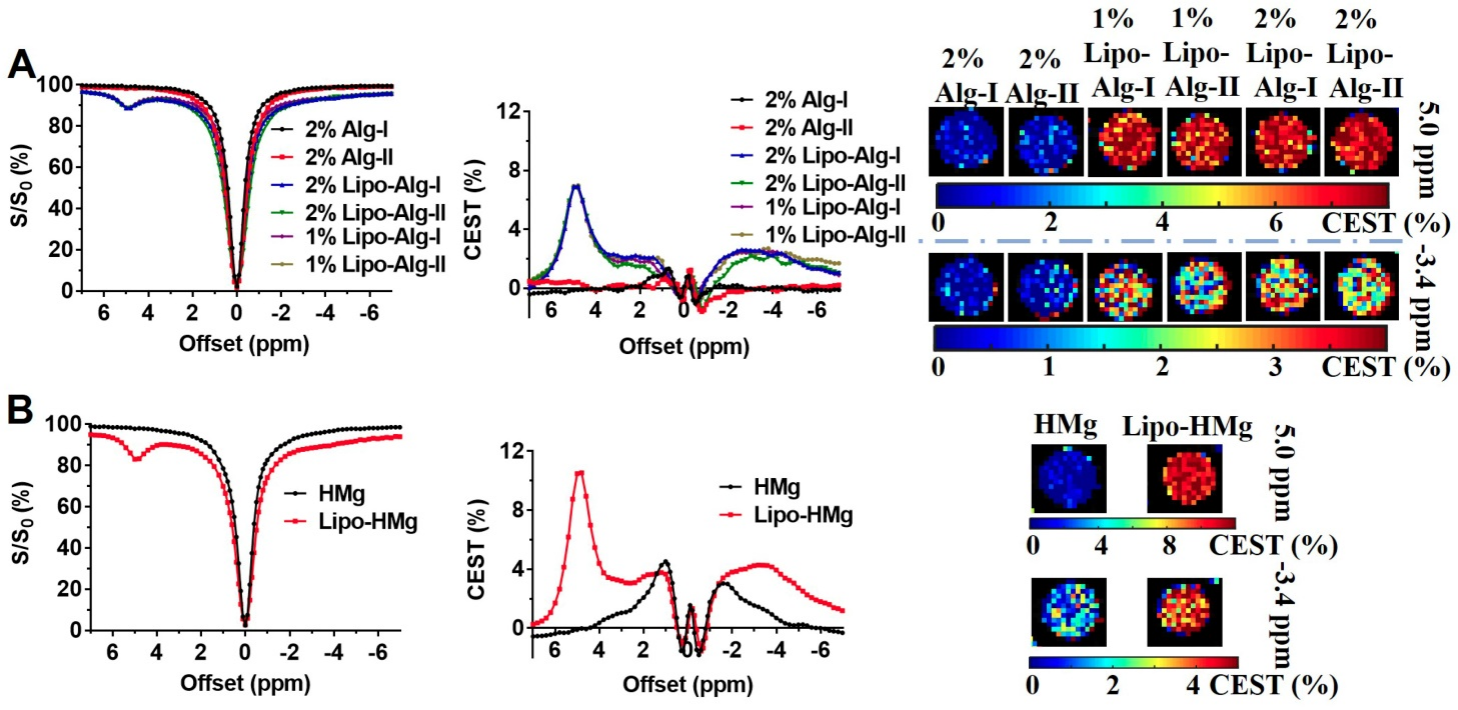
CEST properties of liposomal hydrogels (n=3). **(A)** and** (B)** are Z-spectra, corresponding CEST contrasts, and parametric maps of liposomal alginate and HAMC hydrogels.

As shown in **Figure 4**, both liposomal alginate and HAMC hydrogels generated CEST contrasts at 5.0 ppm and -3.4 ppm and could be applied to semi-quantify the concentration of BA and liposomes, respectively. After the formation of liposomal hydrogels, CEST contrasts were attenuated by the hydrogel preparation as expected. These two distinctive peaks were separated by 8.4 ppm, which enabled the reliable assessment of individual components and facilitated the multiparametric CEST applications 32-35. Alginate liposomal hydrogel (**Figure 4A**) produced CEST contrast of 6.87 ± 0.10% at 5.0 ppm and 2.38 ± 0.28% at -3.4 ppm, whereas HAMC liposomal hydrogel (**Figure 4B**) generated CEST contrast of 10.50 ± 0.06% at 5.0 ppm and 4.27 ± 0.10% at -3.4 ppm. The CEST contrast difference in these two liposomal hydrogels was attributed to the dilution effect during hydrogel preparation. Consequently, the CEST contrast of BAL in liposomal alginate hydrogel was 1.5 times lower than that in liposomal HAMC. Besides, there were two CEST contrasts at 1.0 and -1.4 ppm from the HAMC hydrogel. The contrast at 1.0 ppm corresponded to HA as reported and contrast at -1.4 ppm for MC was first reported here 20-22, 63.

To support the potential application in GBM treatment, we further measured the release profile of gemcitabine (Gem) from our liposomal hydrogels. Gem has a solubility (22.3 mg/mL) comparable to BA (25 mg/mL). It has been applied to treat GBM 64-66. The physiochemical properties of resulting Gem-loaded liposomes were listed in the **Table S2**, showing comparable encapsulation efficiency to BA-loaded liposomes. Furthermore, the cumulative release of Gem (64.28 ± 1.60% over 3 days, **Figure S5**) was comparable to BA (60.89 ± 0.89% at 3 days) from liposomal hydrogel (**Figure S7**). This demonstrated that the liposomal hydrogel we developed in this study is suitable for a sustainable release of Gem in GBM local treatment.

### *In vivo* CEST imaging of liposomal hydrogels

The optimized alginate formulation that we chose for *in* vivo study had both favorable rheological properties of high injectability and being mechanically soft, and a relatively clean background for CEST imaging compared with HAMC. HAMC has additional contrast at 1.0 and -1.4 ppm (**Figure 4B**). As reported previously, soft hydrogel (<300 Pa) could deter cancer cell proliferation and migration 51, 52. We further examined the BA release from relatively soft liposomal alginate hydrogels with comparable storage modulus (**Figure 1B&E**), i.e., 1% Lipo-Alg-II and 2% Lipo-Alg-I. As shown in Figure S5A, both hydrogel formulations showed comparable release profiles, with a slightly lower cumulative release over 3 d in the 2% Lipo-Alg-I formulation. Thus, it was selected and injected into the striatum of the mouse brain for longitudinal monitoring of the drug and liposomes release using CEST contrast at 5.0 and -3.4 ppm at 3T.

**Figure 5:**
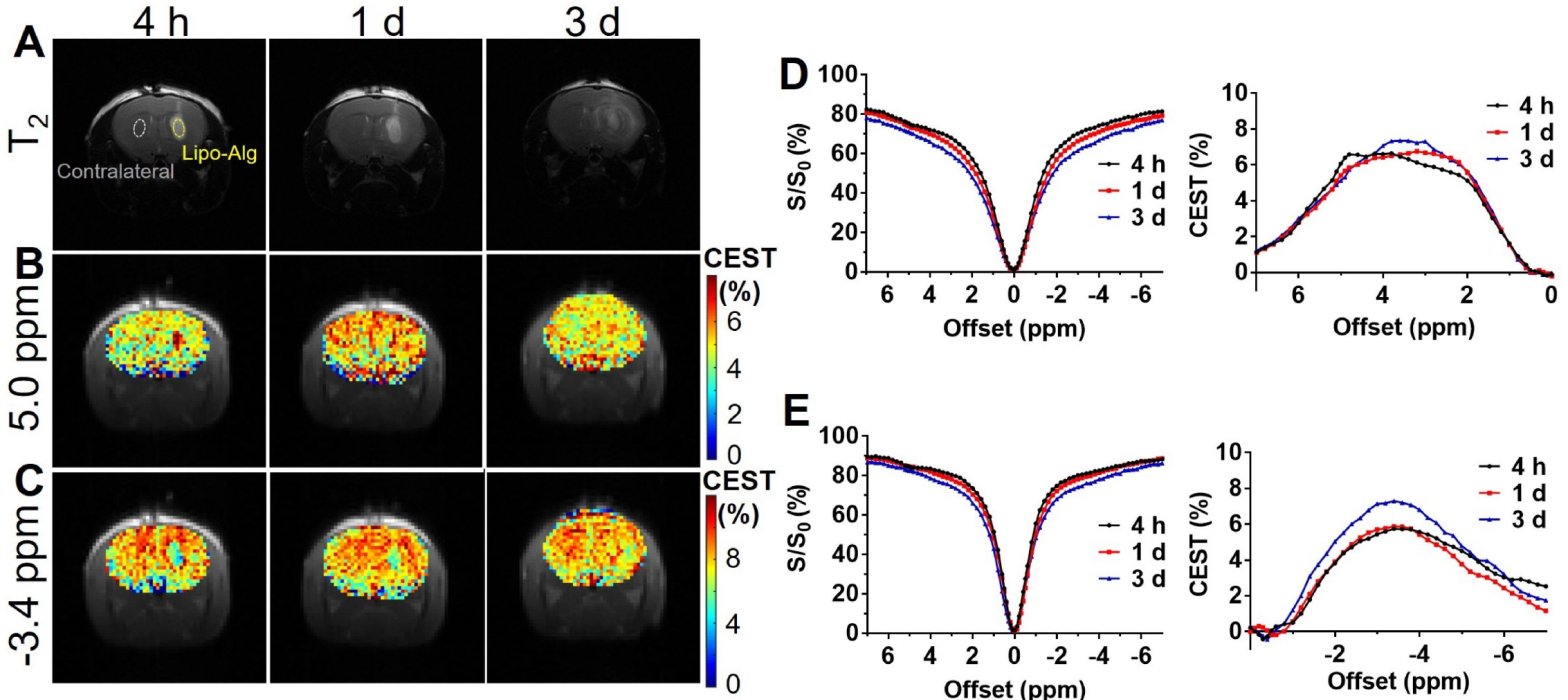
*In vivo* CEST of transplanted Lipo-Alg hydrogel. **(A)** T_2_ anatomical images of hydrogels in the brain; **(B)** and** (C)** CEST maps of the liposomal hydrogel at 5.0 ppm and -3.4 ppm; **(D)** and **(E)** are longitudinal measurements of Z-spectra and corresponding CEST contrast under 1.2 and 0.8 μT, respectively.

**Figure 6:**
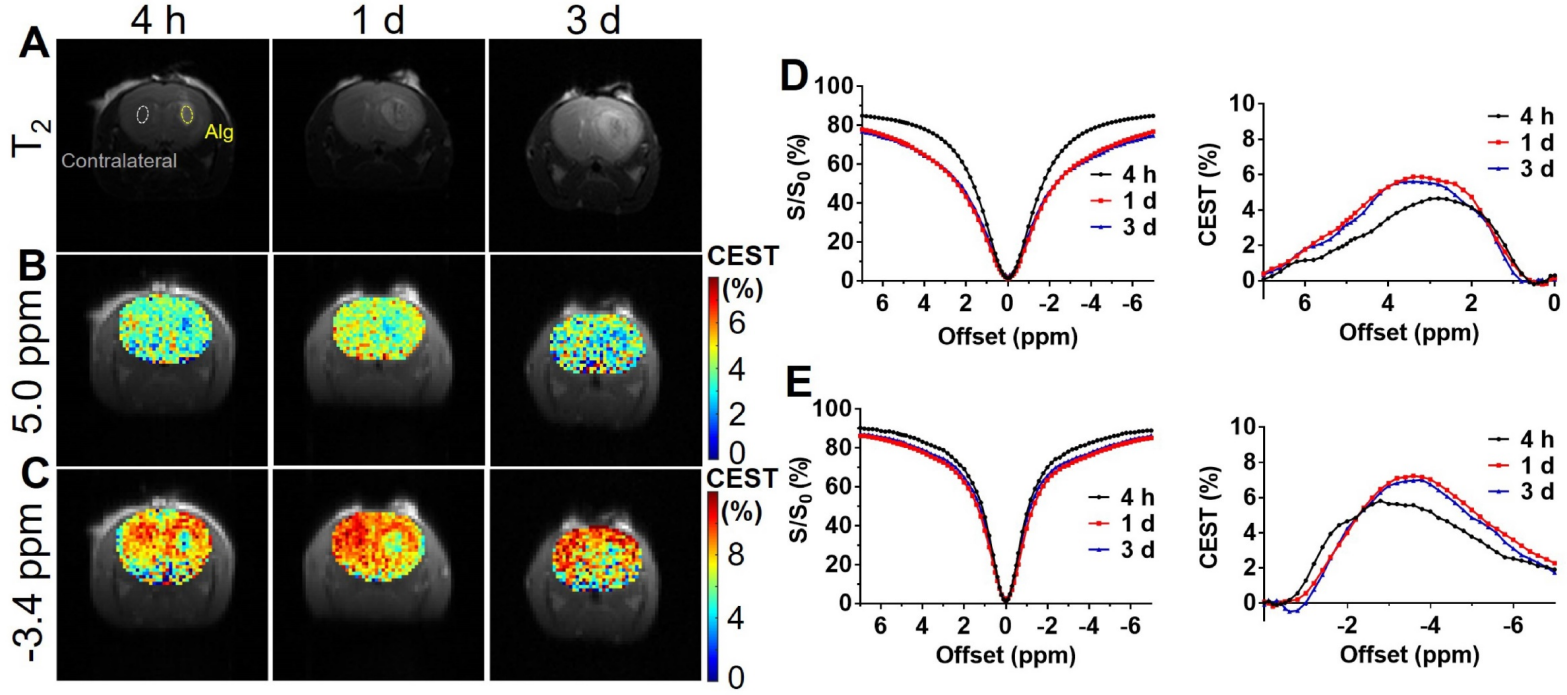
*In vivo* CEST of transplanted alginate (Alg) hydrogel. **(A)** T_2_ anatomical images of hydrogels in the brain; **(B)** and** (C)** CEST maps of Alg hydrogel at 5.0 ppm and -3.4 ppm; **(D)** and **(E)** are longitudinal measurements of Z-spectra and corresponding CEST contrast under 1.2 and 0.8 μT, respectively.

**Figure 7:**
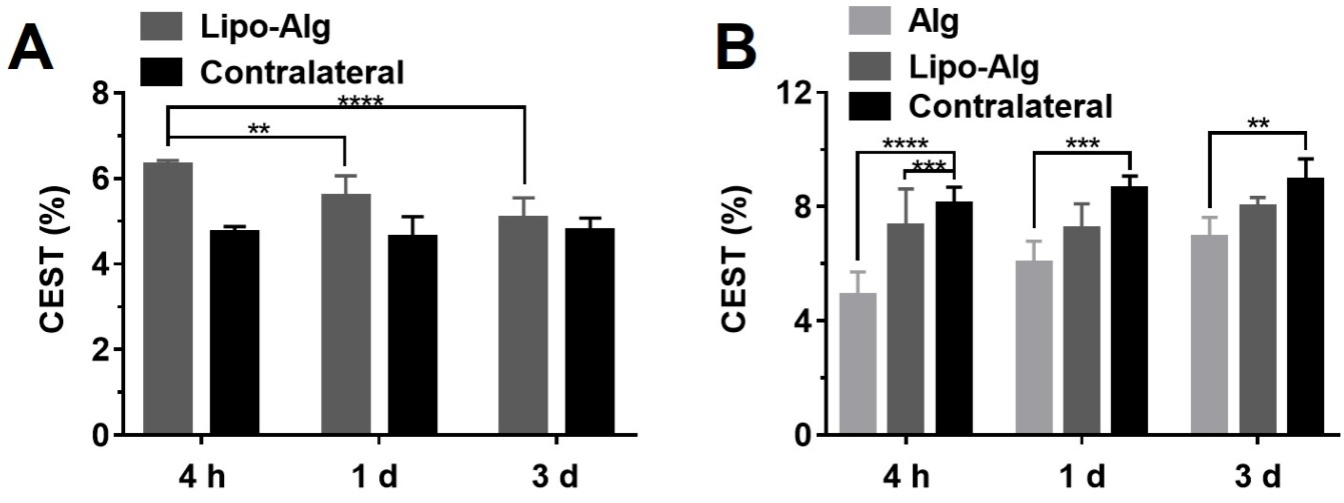
Longitudinal comparison of the CEST contrast of transplanted hydrogels* in vivo*. **(A)** CEST contrast at 5.0 ppm of implanted liposomal hydrogel versus the contralateral region at respective time-points; **(B)** CEST contrast at -3.4 ppm of implanted liposomal hydrogel versus Alg gel and the contralateral region at respective time-points. Significance level was set at **p < 0.01, ***p < 0.001 and ****p < 0.0001 by comparison between the samples at each time point. Values shown are means ± SD (n = 5).

CEST MRI was performed on mouse brain after the injection of liposomal alginate hydrogel (**Figure 5**) and alginate only hydrogel (**Figure 6**). Considering the optimized B_1_ *in vitro* (**Figure 3C**), we further optimized the saturation parameters *in vivo* (**Figure S6**) to minimize other contributions to the Z-spectra, such as direct water saturation (DS) effect and magnetization transfer contrast (MTC). We, therefore, selected 1.2 and 0.8 μT of B_1_ powers for longitudinally monitoring the components of BA and liposomes *in vivo*. As shown in Figures 5 and 7A, the CEST contrast at 5 ppm was 6.32 ± 0.11% higher than the contrast in the contralateral brain (4.74 ± 0.14%) at 4 h post-transplantation. It gradually decreased to 5.07 ± 0.48%, which was comparable to the contralateral region of the brain (4.78 ± 0.30%) over 3 d, which was consistent with the *in vitro* drug release profile (**Figure S5**).

As displayed in Figures 6 and 7B, the liposomal hydrogels (Lipo-Alg) showed CEST contrast of 7.31 ± 1.31% at 4 h after implantation, higher than that of the hydrogel without liposomes (Alg: 4.87 ± 0.84%) at the negative frequency offset (-3.4 ppm). The CEST contrast for Lipo-Alg, Alg, and contralateral side at 3 d post-transplantation were 8.00 ± 0.32%, 6.92 ± 0.35%, and 8.91 ± 0.38%, respectively. Contrary to the decrease of BA contrast at 5 ppm over 3 d, CEST contrast at -3.4 ppm remained constant for Lipo-Alg, demonstrating the different release profiles of the intraliposomal BA and liposomes as nanocarriers. The contralateral side showed the highest and steady CEST contrast of about 8.55 ± 0.46% (**Figure 7B**) in our study, which is consistent with NOE contrast at this saturation 67.

### Histology analysis

**Figure 8:**
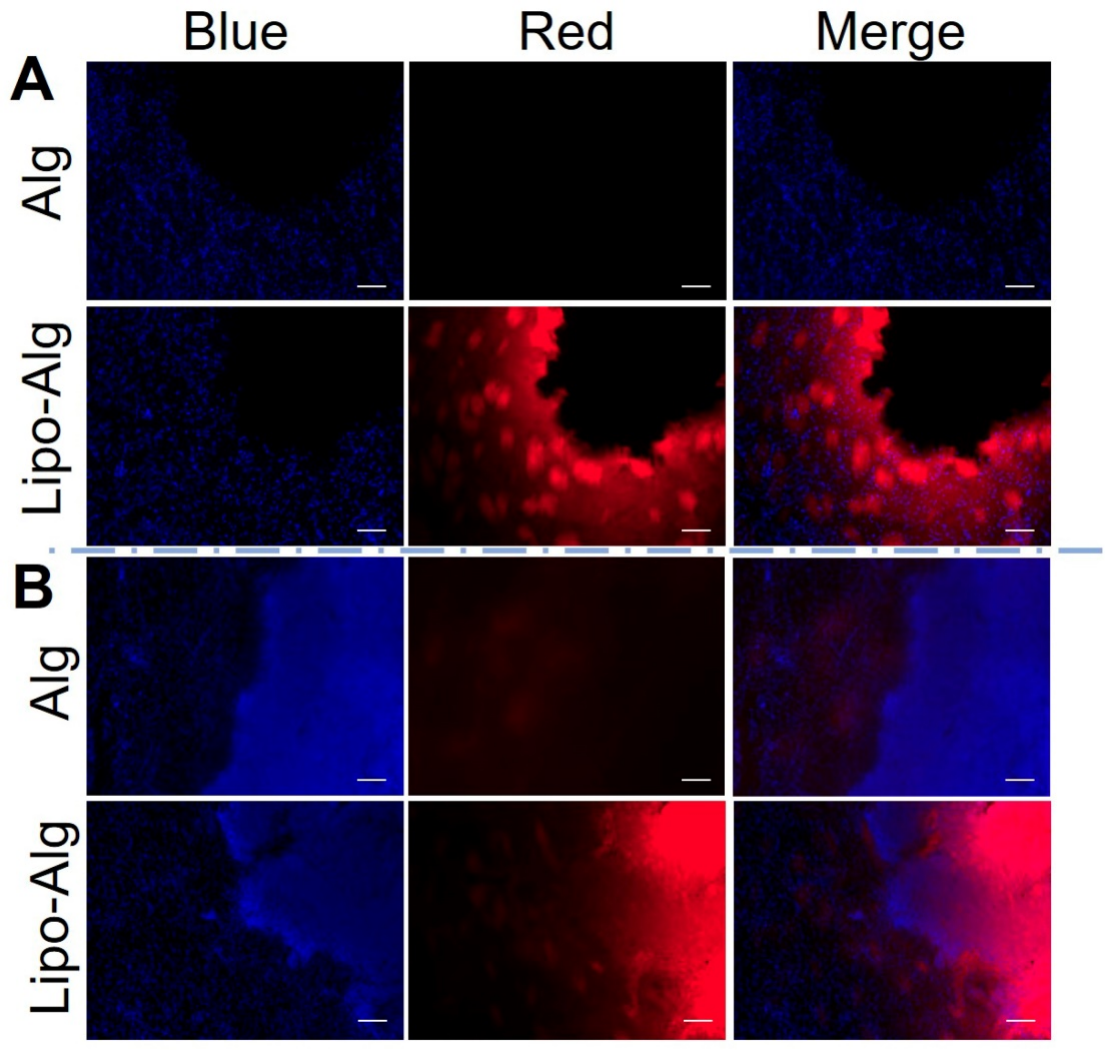
Fluorescence images of the brain tissue slices.** (A)** and **(B)** are fluorescence images after 4 h and 3 d post hydrogel implantation under different channels. DAPI used for cell nucleus staining shows blue fluorescence. The rhodamine B labeled liposomal hydrogel shows red fluorescence. Scale bar = 200 μm.

According to the histology analysis (**Figure 8**), the rhodamine-labeled liposomes in hydrogel were observed at 4 h and up to 3 d post-transplantation. The liposomes were distributed in the periphery of the injection site. The fluorescence intensities were comparable to the retention of liposomes within the injected hydrogel region and were consistent with the constant CEST contrast at -3.4 ppm over 3 d. As compared to the liposomal hydrogel *in vitro* (**Figure S7**), the rhodamine-labeled liposome distribution was comparable to that at 4 h. Moreover, a slight increase in cell density was observed at the periphery of the transplanted region by the nuclear (DAPI) (**Figure 8**) and hematoxylin and eosin (H&E) staining (**Figure S7**). Since cell infiltration could contribute to the CEST contrast at -3.4 ppm *in vivo* 21, 67, we further validated this by imaging a series of cell phantoms with various cell densities dispersed in the same alginate hydrogel. As shown in Figure S8, the NOE contrast was correlated with cell density and became prominent when it was higher than 7.5 × 10^7^ cell/mL. However, the cell density estimated from DAPI staining of the tissues in the hydrogel region was about 5.5 ×10^7^ cell/mL, indicating that the contribution of infiltrated cells to CEST contrast at -3.4 ppm should be minimal. Our histology results further validated the different release profile of drug BA and liposomes from the hydrogel matrix.

## Discussion

We have developed various formulations of CEST MRI detectable hydrogels for monitoring drug delivery to the tumor resection site. The mechanical studies have demonstrated that all hydrogel formulations (**Figures 1** and **2**) are injectable and mechanically soft with storage modulus at 10-250 Pa. The hydrogels showed microporous structures with a pore size of 10-90 μm under SEM. Compared with the structure of alginate (**Figures 1** and **2**), HAMC hydrogels showed increased formation of a fibrous crosslinking network via the hydrophobic entanglement 39, 62, 69, 70. The hydrogel stiffness could be adjusted by both increasing the polymer concentration and crosslinking density while maintaining the injectability. Moreover, incorporation of liposomes into the hydrogel matrices resulted in a slight decrease of the storage modulus (no drastic change in the rheological properties), which could be used to fine-tune the hydrogel mechanical properties. These results support the notion that the liposomal hydrogels enhance drug delivery without compromising their injectability (**Figure 1** and **Figure S1**). Due to their relatively soft (10-250 Pa) composition, the injectable hydrogels are under consideration as an adjuvant treatment for brain cancer. As brain tumors (~26 kPa) are stiffer than normal brain tissue (0.1-1 kPa) 71, 72, glioma cells become rounded, less effective in migration, and less proliferative in the soft hydrogels with storage modulus of 80-250 Pa 51, 52, which is comparable to the softness of our hydrogel formulations. More importantly, our liposomal hydrogels were softer in the presence of liposomes and showed lower brain tumor cell viability as compared to those without liposomes indicating the prospects of our liposomal hydrogels in inhibiting GBM recurrence.

The drug-loaded liposomes showed CEST contrasts at 5 ppm and -3.4 ppm attributed to the intraliposomal barbituric acid (BA) and aliphatic protons of lipids, respectively (**Figure 3**) 15, 36, 37, 73. Both CEST contrasts showed linear relationship with respective concentrations as validated by independent measurements, indicating a promising approach to image both intraliposomal drug and drug nanocarrier semi-quantitively and independently. Furthermore, these two signals were separated over 8 ppm, facilitating the multiparametric imaging at 3T. Previous imaging studies demonstrated that diamagnetic CEST contrast agents separated by a few ppm could be detected at high field strength MRI 32-35. This is even more challenging at clinical field strength (3T), as a large CEST contrast separation is critical to avoid signal overlapping *in vivo*.

Upon incorporation of BAL into the alginate and HAMC hydrogels (**Figure 4**), we consistently observed two distinctive CEST contrasts at 5 ppm and -3.4 ppm *in vitro* (n=3). This represents a robust approach to design and prepare CEST imageable hydrogels*.* These promising findings indicate that multiparametric CEST imaging could be used for independent and simultaneous monitoring and measurement of the drug (5.0 ppm) and liposome (-3.4 ppm) release following transplantation of the hydrogels into the mouse brain. Furthermore, the HAMC hydrogels showed concurrent CEST contrasts at 1.0 ppm and -1.4 ppm ascribed to the hydroxyl groups of HA and the methoxy protons of methylcellulose. The CEST contrast at -1.4 ppm corresponds to the peaks of methylcellulose at 3.5 and 3.3 ppm (with respect to tetramethylsilane) in the ^1^H-NMR spectrum 20-22, 63. These additional contrasts of HAMC hydrogels were suitable for monitoring of hydrogel matrix after transplantation not only for the degradation of the whole matrix but also of individual components.

Our study aimed to develop CEST MRI detectable hydrogel as the delivery vehicle instead of the wafer because the side effects of Gliadel originating from the wafer have hampered its therapeutic efficacy 5,8,74,75. Moreover, a softer hydrogel with a storage modulus of 10-300 Pa is comparable to the normal brain tissue (0.1-1 kPa) and better suited to minimize cancer cell migration 51, 52, 76, which was also demonstrated in our study (**Fig. S3**). Another advantage is that the soft hydrogel provides better coverage in the resection cavity than the rigid wafer. Currently, there is no imaging approach to characterize the delivery vehicle directly. Conventional MRI contrast, such as T_1_ and T_2_, could be applied but do not provide sufficient information to guide treatments 77-80. Our optimized soft and injectable hydrogel can address these issues and has multiple CEST contrasts to measure the drug and its carrier liposomes at clinical field strength (3T).

After injection of the optimized hydrogel into the striatum of the mouse brain, we observed the CEST contrast at 3T for the intraliposomal drug at 5 ppm and for liposomes at -3.4 ppm (**Figures 5** & **6**). CEST contrast at 5 ppm represented the relative concentration of intraliposomal BA and CEST contrast at -3.4 ppm was indicative of the aliphatic protons of lipids in the liposomal hydrogel, i.e., the relative concentration of liposomes (**Figure 3**) 15, 36, 37, 73. This optimized formulation (i.e., 2% Lipo-Alg-I) had a slower drug release than 1% Lipo-Alg-II and was capable of carrying BA and anticancer drugs (e.g., Gemcitabine, **Table S2** and **Fig. S5**) with a release comparable to *in vitro* 64-66, 81, 82*.* CEST contrast at 5 ppm decreased while the CEST contrast at -3.4 ppm was relatively constant throughout the study, which indicated a different rate of release of these components *in vivo.* CEST contrast at -3.4 ppm of liposomal hydrogel was consistently higher than that of alginate hydrogel, which indicated the uniqueness of this contrast for monitoring liposomes as confirmed by histology (**Figure 8** and **S7**). CEST contrast at -3.4 ppm could also originate from aliphatic protons of the cell membrane *in vivo*, for instance, from the contralateral brain. To further illustrate its specificity for the detection of liposomes, we examined the CEST contrast at -3.4 ppm at various cell densities *in vitro* (**Figure S8**). An increase in the number of cells contributed to the contrast at -3.4 ppm and 2-4 ppm. It is estimated that this contribution would be around 1% due to the cell density in the brain. This also provides an explanation for the slightly higher CEST contrast at -3.4 ppm on day 3 as compared to that 4 h after injection. Nevertheless, our findings demonstrated that both the drug and liposomes release could be monitored by CEST MRI independently and simultaneously *in vivo* at 3T.

We used Lorentzian fitting for analyzing the z-spectrum *in vivo,* which fitted the raw data well (Figure S9). At the B_1_ used in our study, we did not observe major contributions from other endogenous exchanges within the liposomal hydrogel over the period of our study. This power level was also recently reported to have minimal contributions from endogenous contrast, such as amide proton transfer (APT) and NOE 83. Thus, the longitudinal CEST contrast changes should be mainly caused by the different release profiles of BA and liposomes. Also, there are numerous approaches for analyzing the z-spectrum for CEST contrast characterization, such as MTR_asym_ and multi-pool Lorentzian fitting 83, 84. Depending on the application and contributions from endogenous contrast, these approaches should be carefully selected to highlight the underlying exchange mechanisms *in vivo*.

## Conclusion

The liposomal hydrogels, especially MRI-detectable hydrogels, are very promising for controlled drug release and local treatment. In this study, we developed injectable liposomal hydrogels with multiparametric CEST contrasts at 3T using the clinical-grade alginate and HAMC hydrogels. All formulations were injectable and mechanically soft and, therefore, were suitable for delivering chemotherapeutics to the brain and deterring cancer cell proliferation. After transplantation into the mouse brain, the model drug BA and liposome release could both be monitored by CEST at 5.0 and -3.4 ppm over 3 d. The longitudinal changes of independent CEST contrast reflected different release profiles, which could be validated by histology analysis. This multiparametric imaging approach allowed simultaneous and independent monitoring of both the drug and the liposome carrier in the hydrogel matrix. Besides the degradation of the hydrogel matrix, our approach provides a comprehensive readout for compositional change in hydrogel-based therapy, allowing image-guided therapy to refine treatments.

## Materials and Methods

**Materials:** 1,2-dipalmitoyl-snglycero-3-phosphocholine (DPPC) and 1,2-distearoyl-sn-glycerophosphoethanolamine poly (ethylene glycol) 2000 (DSPE-PEG-2000) were obtained from the Acanti Polar Lipids, Inc. (Alabaster, AL). Barbituric acid (BA), calcium D-gluconate, methylcellulose (MC, M0512, 4000 cP), cholesterol, Triton X-100, and culture flasks (Corning® T-25) were purchased from Sigma-Aldrich (St. Louis, MO). Sodium hyaluronate (HA15M) was purchased from Lifecore Biomedical Inc., Chaska, MN. Sephadex G50 columns were bought from GE Healthcare Life Sciences, Pittsburg, PA. Dulbecco's Minimum Essential Medium (DMEM, GlutaMAX^TM^-1), phosphate-buffered saline (PBS, pH 7.4), fetal bovine serum (FBS), penicillin-streptomycin and trypsin were all purchased from Gibco, Invitrogen. Cell Counting Kit-8 (CCK-8) was purchased from Dojindo Laboratory (Dojin, Japan).

**BA-Liposome (BAL) preparation:** Liposomes were prepared using the thin-film hydration method 85, 86. It was composed of lipids DPPC, cholesterol, and DSPE-PEG2000 at a molar ratio of 10:8:1. In brief, the lipid mixture was dried on a rotary evaporator to form a homogeneous thin film layer with lipid weights of 25, 50, and 75 mg. Subsequently, 1 mL BA solution (25 mg/mL, pH 7.2) was added to hydrate the thin film under 60^ o^C for 1 h. For the preparation of gemcitabine-(Gem) loaded liposomes or empty liposomes, 1 mL Gem solution (22.3 mg/mL, pH 7.2) or water was added for hydration, respectively. The resultant mixture was sonicated to form a lamellar liposome solution, which was further extruded through 400 nm polycarbonate filters with an Avanti Mini-Extruder (Alabaster, AL) to obtain liposomes with targeted size. The un-encapsulated BA was removed through a Sephadex G50 column twice. The liposome stock solution was stored at 4 ^o^C before use.

**Liposome characterization and BA loading determination:** The size, polydispersity index (PDI) and surface charge of liposomes were measured by dynamic light scattering (DLS) at room temperature by Zetasizer (Malvern Instruments, UK). The particle concentration was measured by Nanosight (Malvern Instruments, UK).

The loading of BA and Gem in liposomes was determined by measuring the UV absorbance at 257 nm and 268 nm using UV-VIS spectrometer (PerkinElmer Lambda 35), respectively. BA liposomes were treated with Triton X-100 solution to completely release the BA payload, diluted to proper concentration, followed by UV measurements. The concentration was then determined by the calibration curves of BA solutions with known concentrations.

**Alginate hydrogel preparation:** Alginate hydrogels with variable concentrations and percentages of crosslinking density were prepared according to the following procedures. Alginate solution with 1 or 2 wt% concentration was mixed with an equal volume of liposomes or water for the control. The resultant mixture was then mixed with equivalent calcium gluconate solution of 0.3, 0.6, and 1.2 wt% concentration. The formulations were abbreviated as 1% Alg-I, 1% Alg-II, 2% Alg-I, 2% Alg-II, 1% Lipo-Alg-I, 1% Lipo-Alg-II, 2% Lipo-Alg-I, and 2% Lipo-Alg-II, where 1% and 2% represent the alginate concentration; Alg-I and Alg-II represent the alginate hydrogel with 40% or 80% crosslinking, respectively.

**HAMC hydrogel preparation:** Methylcellulose polymer solution was prepared by a dispersion technique 87. Briefly, the one-half volume of water was heated to 90^ o^C and 1.5 wt% of MC powder was added and agitated until all polymers were thoroughly wetted. The remaining half of water was then added, gently stirred at room temperature, and equilibrated at 4 ^o^C overnight to obtain a clear solution. Sodium hyaluronate (HA15M) solution was directly prepared in water and equilibrated at 4^ o^C overnight. HAMC hydrogels was prepared through the physical blending of equal volumes of HA and MC. The resulting mixture was homogenized using a three-way stopcock connected with two syringes and centrifuged to remove bubbles. The prepared hydrogel was stored at 4^ o^C before use. As for the liposomal HAMC, the HA was dissolved in the liposome solution before mixing it with MC. The hydrogel formulations were abbreviated as 0.75% HMg and 0.75% Lipo-HMg, where 0.75% denoted the final concentration of HA and MC.

**Hydrogel mechanical and morphology studies:** All rheological measurements were performed on a KINEXUS Pro+ (Malvern, UK) rheometer using a parallel-plate configuration with a 20 mm diameter (gap: 0.2 mm). Dynamic oscillatory frequency sweeps were conducted from 1 to 100 Hz at a 1% strain amplitude after equilibration for 2 min at 37 ^o^C to determine the mechanical properties of the alginate hydrogels. Shear-thinning properties of the hydrogel were characterized by measuring linear viscosity (η) under a time sweep mode at alternating low and high shear rates of 1 to 100 s^-1^ at room temperature (25 ^o^C). All measurements were performed at least 3 times per formulation.

The morphology of the hydrogel was observed by FEI Quanta 250 Environmental SEM. Samples were placed on a clean glass, frozen in liquid nitrogen, and freeze-dried overnight. The prepared samples were coated with a thin layer of gold by QUORUM #Q150TS dual-target sputtering system before SEM observation. The pore size was analyzed by Image-J.

**Hydrogel cytocompatibility:** U-87 MG glioma cells (ATTC, USA) were cultured in DMEM supplemented with 10% FBS and 1% penicillin-streptomycin. Cells were cultured in culture flasks (T-25) and incubated at 37 °C and 5% CO_2_. To study the cytocompatibility of hydrogel formulations, U-87 MG glioma cells were first seeded in 96-well (10,000 cells/well) plates. The culture medium was changed to fresh one after overnight, and 50 μL of hydrogel was added to each well; PBS was added to the control well. Cell viability was determined by the Cell Counting Kit-8 (CCK-8) assay after two days in culture and quantified by UV-absorption at 450 nm using a microplate reader (Spectramax M5e). The number of viable cells was normalized to the PBS-treated control group (n = 8 per sample).

**Phantom preparation and drug release:** The BAL with 75 mg/mL concentration was used to prepare hydrogel samples. All liposomal hydrogel formulations were prepared as described above. For the control hydrogel phantom, the hydrogel was mixed with water instead of the BAL solution. All hydrogels were centrifuged at 3000 rpm to remove bubbles before MRI measurements. The Gem-loaded liposomal alginate hydrogel was prepared similar to BA-loaded liposomal hydrogel. First, the BA release from liposomal hydrogel with a formulation of 1% Lipo-Alg-II and 2% Lipo-Alg-I was studied by adding 200 μL hydrogel with 1.8 mL aCSF buffer to an Eppendorf tube at 37 ^o^C 69. At each time point, 200 μL supernatant was removed and replaced with fresh aCSF. Gem released from the optimized 2% Lipo-Alg-I was also measured in the same fashion. After sonication at 45 ^o^C for 10 min, UV absorbance was measured at 257 nm and 268 nm for BA and Gem, respectively.

**CEST imaging protocol:** BAL and liposomal hydrogels was imaged at a horizontal bore 3T preclinical Bruker MRI system (Bruker, Ettlingen, Germany) using a 3s continuous-wave (CW) saturation pulse at 37 ^o^C by a gas warming system equipped with a 40 mm volume coil transmitting and receiving. Samples in a 6 mm glass tube or 0.5 mL tube were placed parallel to the magnet field. The B_0_ field was shimmed to the second-order using water linewidth. A modified rapid acquisition with relaxation enhancement (RARE) sequence, including a saturation image-guided pulse, was used to acquire CEST images at different irradiation frequencies, which were used to generate the Z-spectrum in each voxel. Images were acquired with the following parameters: slice thickness = 1 mm, field of view (FOV) = 20×20 mm, image size = 64×64, RARE factor = 32, repetition time/echo time (TR/TE) = 5000/4.7 ms with 10.45 s acquisition time for every offset and a total 930 s for a full Z-spectrum. Z-spectra were acquired at varying saturation pulse (B_1_) amplitudes including 0.6, 0.8, 1.0, 1.2 and 1.4 μT with 3000 ms saturation duration (T_sat_) to optimize the saturation parameters. The frequency offsets were set from -7 to 7 ppm, with 0.2 ppm or 25.6 Hz step size, around the water resonance (0 ppm). Water Saturation Shift Referencing (WASSR) was also acquired for water frequency correction 88 using the same parameters except for a saturation pulse length of 500 ms, a saturation field strength (B_1_) of 0.2 μT with frequency offsets from -1.0 to 1.0 ppm (step size = 0.1 ppm). Finally, 0.8 μT B_1_ field strength, 3000 ms T_sat_ and 5000 ms TR were chosen for subsequent liposome and liposomal hydrogel studies.

The data were processed using custom-written MatLab (Mathworks, Natick, MA) scripts with the CEST contrast quantified by calculating from the mean of an ROI placed over each sample after B_0_ correction of the contrast on a pixel-wise basis. The water direct saturation (DS) was removed by assuming it as a Lorentzian function using the Z-spectrum between -0.8 ppm to 0.8 ppm and the signals between 6~7 ppm. The CEST contrast (%) was quantified by subtracting the Z-spectra from the Lorentzian fitted water signal 37.

**Hydrogel implantation and *in vivo* CEST imaging:** To increase the contrast of liposomal hydrogel and facilitate *in vivo* monitoring, the BA-liposomes were lyophilized and rehydrated with 1% alginate solution overnight. All solutions were sterilized under UV exposure for 30 min before gelation. Subsequently, an equal volume of 0.4 wt% calcium gluconate solution was added, agitated, and homogenized by pipetting up and down.

Female NOD-SCID mice (6~8 weeks) were acquired from the laboratory animal services center of the Chinese University of Hong Kong. All experimental animal procedures complied with the regulation of Animals (Control of Experiments) Ordinance (Chapter 340, Department of Health, Hong Kong) and had been approved by the Animal Experimentation Ethics Committee of the University. The mice were housed in the Laboratory Animal Research Unit of the City University of Hong Kong under a pathogen-free condition with free access to food and water.

The mice were anesthetized using 1.5-2.5% isoflurane in oxygen at 1.5 L/min, positioned on a stereotaxic device, and maintained by isoflurane gas anesthesia. Five μL of alginate hydrogel with or without BA-liposomes was injected into the brain by a Hamilton airtight syringe (10 μL) under vaseline seal with the flow rate of 0.2 μL/min and the coordination of 0.2 mm anterior, 2.2 mm lateral from the bregma and 3.9 mm deep. The needle was kept in the position for 10 min and slowly withdrawn at a rate of 0.5 mm per 2 min. Animals were imaged at 4 h, 1, and 3 d post-implantation using a 3.0 T Bruker MRI system (Bruker, Germany) after anesthetization with isoflurane in oxygen (1.5-2.5% for induction and 1% for maintenance). A quadrature coil of 82 mm diameter and a 23 mm diameter mouse brain surface coil were used for the transmitting and receiving signal, respectively. Respiration was continuously monitored by a pneumatic pillow sensor and respiration monitoring system. The warming pad was attached to the mouse back to keep the body temperature at 37 ^o^C.

Shimming up to second order was performed using a mouse brain field map before anatomical and CEST acquisition. T_2_ weighted images were acquired using a RARE sequence (TR = 2500 ms; TE = 54 ms; RARE factor = 16; FOV = 20 × 20 mm; image size = 128 × 128) to determine the hydrogel location and select slice for CEST imaging. The same WASSR, CEST sequences and parameters used in *in vitro* studies were applied for *in vivo* imaging. After optimization with a series of B_1_ powers, 1.2 and 0.8 μT were selected for longitudinally monitoring BA and NOE contrast, respectively. The data processing method was the same as *in vitro* analysis.

**Histology analysis:** Animals at 4 h and 3 d after transplantation were anesthetized and perfused with PBS and 10% neutral buffered formalin to fix brain tissues. Subsequently, the brain tissues were dissected, post-fixed in 10% formalin over two days, then transferred to 30wt% sucrose solution and kept at 4 ^o^C. Histological sections were cut on a cryostat (Leica) with 40 μm thickness and directly mounted onto positively charged microscopic slides. Histological analysis was performed by H&E staining according to the standard protocols. For DAPI staining, the tissue slices were directly mounted onto slides using mounting solution with DAPI (ProLong® Gold Antifade Mountant, Thermo). Microscopic and fluorescence images were acquired with an Olympus BX40 microscope (Olympus, Tokyo, Japan).

**Statistical Analysis:** Statistical significance was evaluated with Prism 6 (GraphPad Software). Comparisons were made between the groups of mice at several time points using two-way ANOVA. Differences were considered as statistically significant for a P-value <0.05.

## Supplementary Material

Supplementary figures and tables.

## Figures and Tables

**Table 1:** Physicochemical properties of various BA-liposome (BAL) formulations.

Lipid Conc. (mg/mL)	Size (nm)	PDI	Z-potential (mV)	BA Conc. (mg/mL)	Encapsulation Efficiency (%)
25	206.3±1.2	0.121±0.028	-0.47±0.17	13.57±0.43	54.28±1.72
50	196.1±0.7	0.115±0.004	-0.83±0.26	15.48±0.22	61.92±0.88
75	214.6±1.2	0.211±0.007	-0.80±0.24	19.21±0.35	76.84±1.40

Data represent mean ± S.D. (n ≥ 3).
