# Supplementary material for: CEST MRI detectable liposomal hydrogels for multiparametric monitoring in the brain at 3T
Source: Theranostics. 2020 Jan 12;10(5):2215–28. doi: 10.7150/thno.40146 (PMC7019148; doi:10.7150/thno.40146)
Supplement: Supplementary file 1 — Supplementary figures and tables. [file thnov10p2215s1.pdf]

Supporting Information for:

CEST MRI detectable liposomal hydrogels for multiparametric monitoring in the brain at 3T

Xiongqi Han[1], Jianpan Huang[1], Anthea K.W. To[3], Joseph H.C. Lai[1], Peng Xiao[1], Ed X. Wu[3], Jiadi Xu[2,4], Kannie W.Y. Chan[1,2]*

[1]Department of Biomedical Engineering, City University of Hong Kong, Kowloon Tong, Hong Kong;
[2]Russell H. Morgan Department of Radiology and Radiological Science, The Johns Hopkins University School of Medicine, Baltimore, USA;
[3]Department of Electrical and Electronic Engineering, The University of Hong Kong, Pokfulam, Hong Kong.
[4]F.M. Kirby Research Center for Functional Brain Imaging, Kennedy Krieger Institute, Baltimore, USA.

Corresponding author:

Kannie WY Chan,
City University of Hong Kong
83 Tat Chee Ave.,
Kowloon Tong, Hong Kong
Tel: +852 34429141
Email: KannieW.Y.C@cityu.edu.hk

Keywords: CEST MRI, hydrogel, liposome, glioblastoma

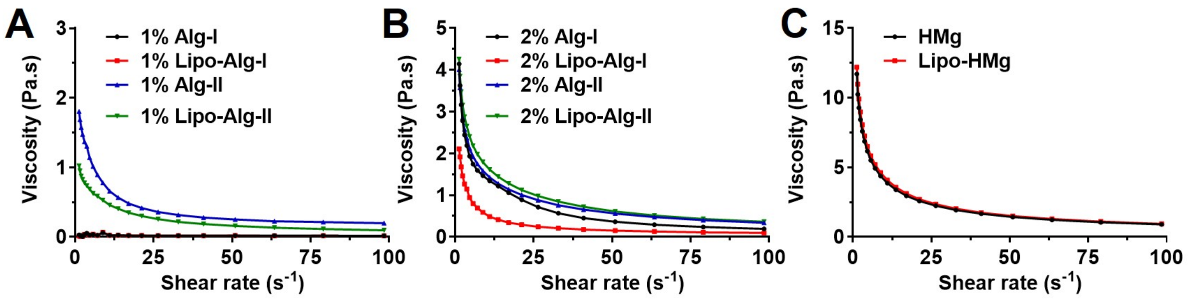

Figure S1. Injectabilities of hydrogels. **(A)** and **(B)** were viscosity measurements of 1 wt% and 2 wt% alginate hydrogels with and without liposomes under 40% and 80% crosslinking density under various shear rate; **(C)** was viscosity measurements of 0.75% HAMC hydrogels with and without liposomes under various shear rate.

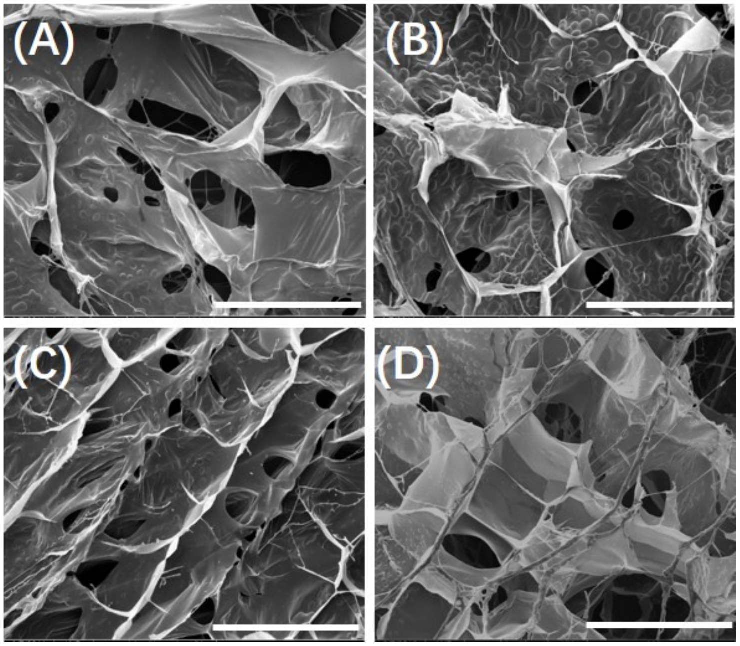

Figure S2. SEM images of the liposomal hydrogel. **(A)** and **(B)** were 1 wt% liposomal alginate hydrogel with 40% and 80 % crosslinking; **(C)** and **(D)** were 2 wt% liposomal alginate hydrogel with 40% and 80 % crosslinking. Scale bar=200 μm.

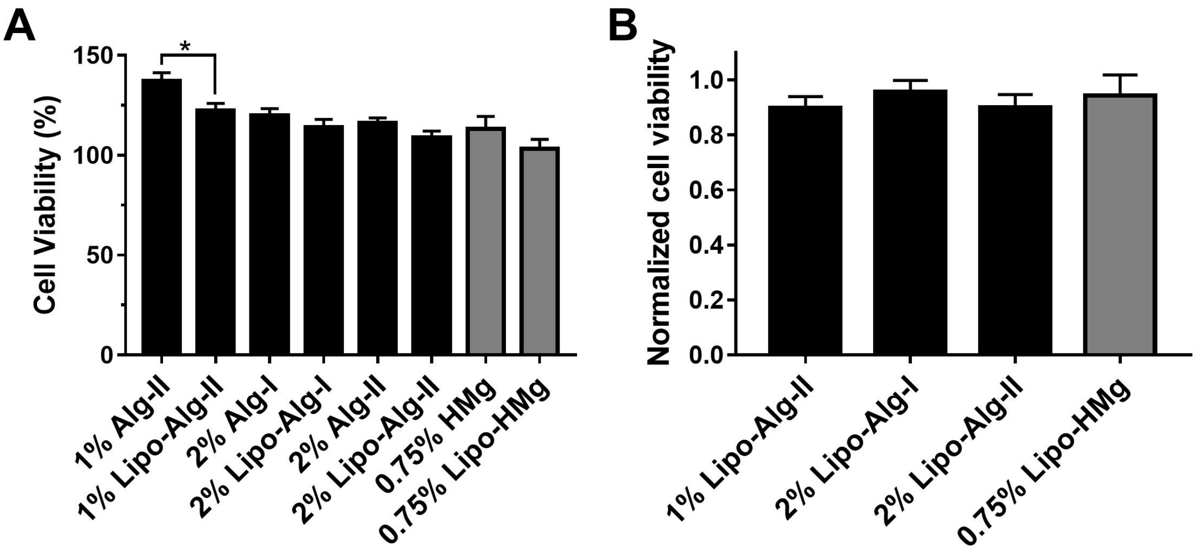

Figure S3. Cell viability of hydrogels without and with liposomes in **(A)** percentage; and **(B)** in normalized cell viability with reference to hydrogels without liposomes (*n = 8)* two days in culture, where 1% and 2% were the alginate concentration; Ag-I and Ag-II were the alginate hydrogel with 40% or 80% crosslinking density; HMg was the abbreviation of HAMC hydrogel; Lipo represented the liposome-containing hydrogel.

Table S1. The CEST contrast (%) at 5.0 and -3.4 ppm in different BA-Liposome concentration under 0.8 μT.

**Lipid Conc. (mg/mL)**	**Liposome Conc. (particles/mL)**	**CEST (%) -3.4 ppm**	**BA Conc. (mg/mL)**	**CEST (%) 5.0 ppm**
0	0	0	0	0
25	1.01×10^{16}	5.94	13.57	20.76
50	1.54×10^{16}	10.30	15.48	25.75
75	1.70×10^{16}	13.20	19.21	26.66

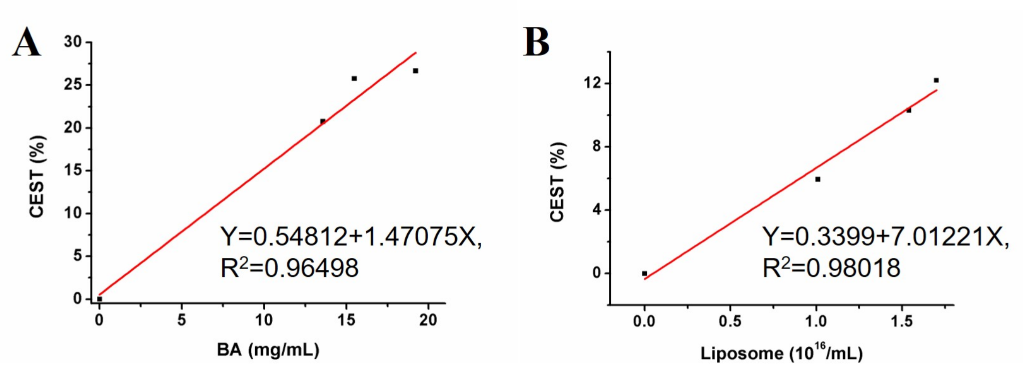

Figure S4. The linear fitting of CEST contrast at 5.0 ppm **(A)** and -3.4 ppm **(A)** by using the data listed in **Table S-1**.

Table S2. Physicochemical properties of Gemcitabine loaded liposomes.

**Lipid Conc. (mg/mL)**	**Size (nm)**	**PDI**	**Z-potential (mV)**	**Gem Conc. (mg/mL)**	**Encapsulation Efficiency (%)**
25	218.2±3.9	0.127±0.032	-0.79±0.81	14.87±1.27	50.80±0.79

Data represent mean ± S.D. (n ≥ 3).

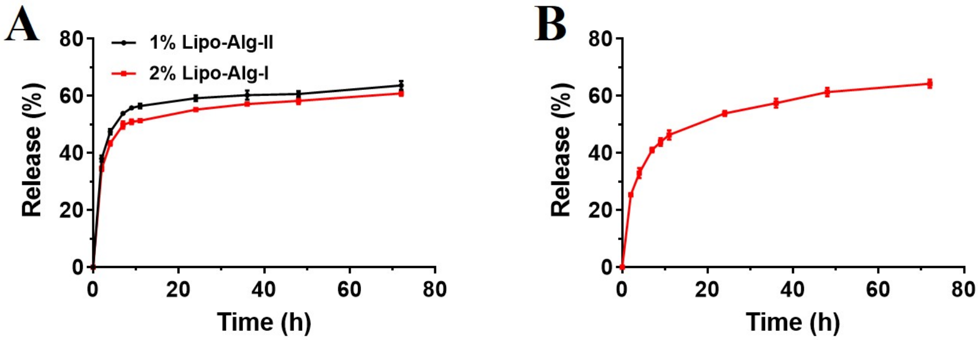

Figure S5. Drug cumulative release from liposomal hydrogel. **A** BA release profile from the liposomal hydrogel of 1% Lipo-Alg-II and 2% Lipo-Alg-I, which showed equivalent mechanical properties. **B** Gemcibine (Gem) release profile from its liposomal hydrogel of 2% Lipo-Alg-I. Data was presented as mean ± SD (n=3).

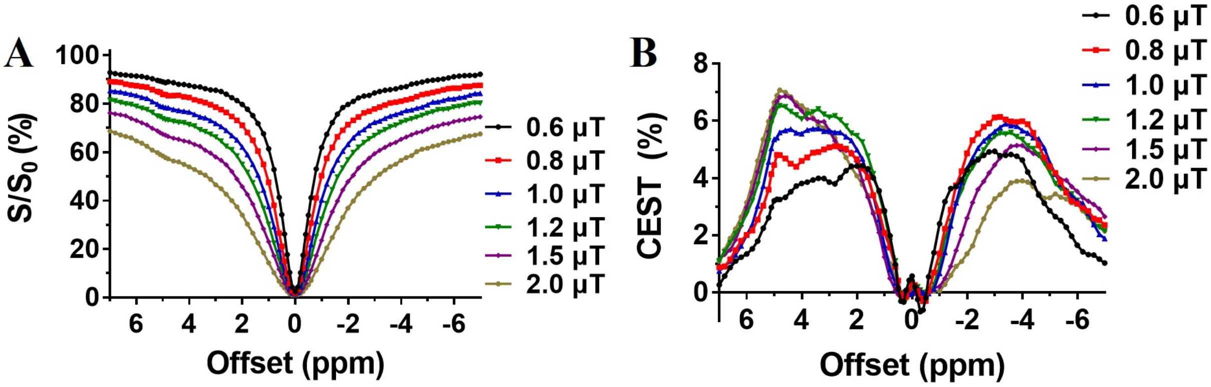

Figure S6. B_1 optimization for *in vivo* measurements. **A** and **B** were the Z-spectra and corresponding CEST contrasts of liposomal hydrogel 4 hours after transplantation under various B_1 fields. Under 0.8 μT, it gave out the highest NOE contrast at -3.4 ppm. While under 1.2 μT, it produced good CEST contrast with clear peaks at 5.0 ppm. Thus, 0.8 μT and 1.2 μT were selected for *in vivo* monitoring of CEST contrast at -3.4 and 5.0 ppm, respectively.

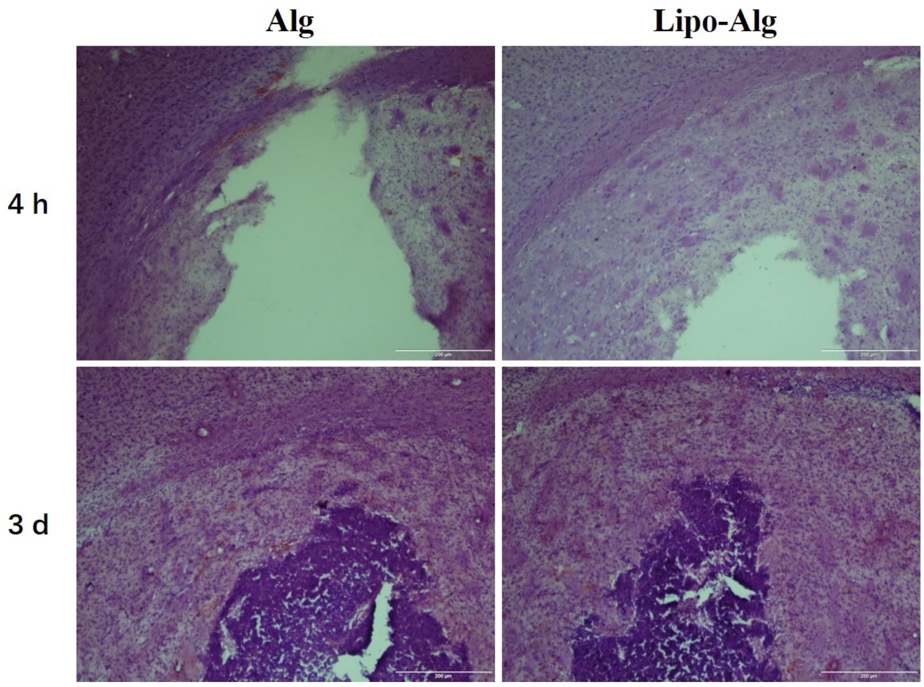

Figure S7. Histological assessment 4 hours and 3 days after hydrogel implantation using H&E staining, in which the scaffold appeared purple. Scale bar = 200 μm.

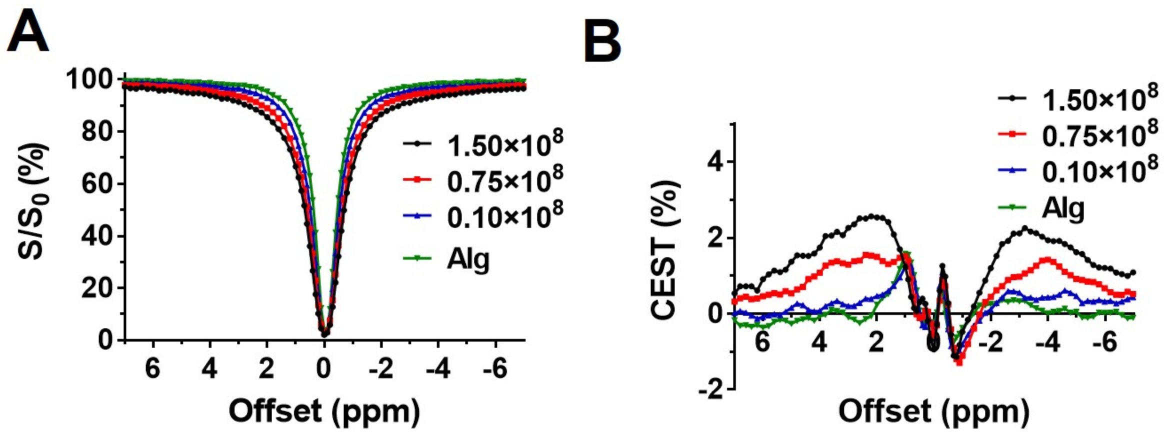

Figure S8. CEST contrast of U87 cell dispersed in alginate hydrogel with various cell density. **A** Z-spectra and **B** the corresponding CEST contrast by Lorentz fitting.

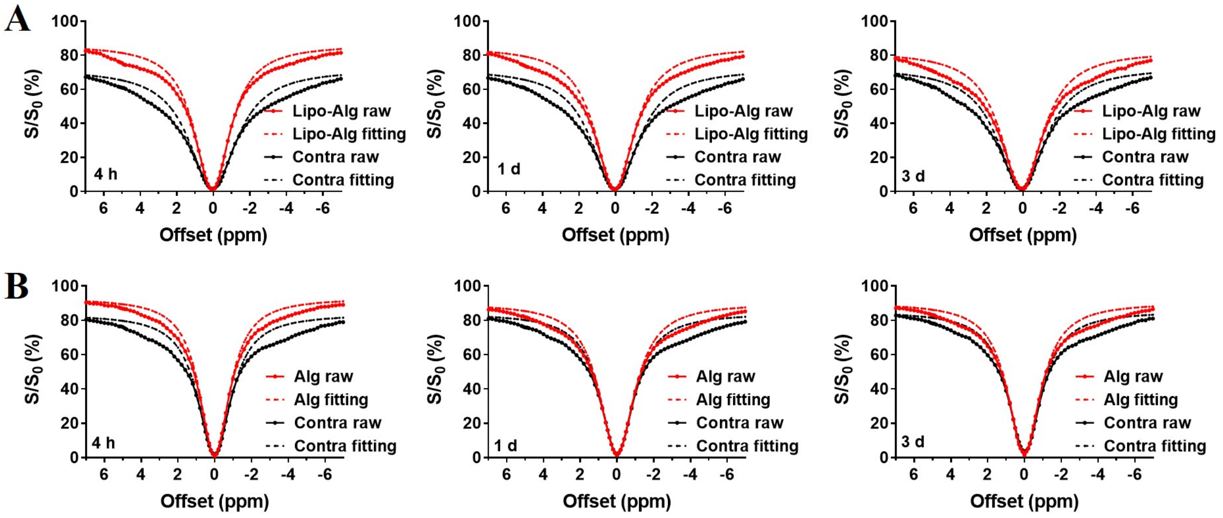

Figure S9. The *in vivo* representative longitudinal Z-spectra raw data and Lorentzian fitting results using the Z-spectra between -0.8 ppm to 0.8 ppm and 6~7 ppm. **(A)** and **(B)** were respectively for Lipo-Alg under 1.2 μT and Alg hydrogel 0.8 μT at different time points.
